# Phase Locking Asymmetries at Flexor-Extensor Transitions during Fictive Locomotion

**DOI:** 10.1371/journal.pone.0064421

**Published:** 2013-05-21

**Authors:** David L. Boothe, Avis H. Cohen, Todd W. Troyer

**Affiliations:** 1 Neurosciences and Cognitive Sciences Program, University of Maryland at College Park, College Park, Maryland, United States of America; 2 Department of Biology, University of Maryland at College Park, College Park, Maryland, United States of America; 3 Institute for Systems Research, University of Maryland at College Park, College Park, Maryland, United States of America; 4 Department of Biology, University of Texas at San Antonio, San Antonio, Texas, United States of America; Emory University, United States of America

## Abstract

The motor output for walking is produced by a network of neurons termed the spinal central pattern generator (CPG) for locomotion. The basic building block of this CPG is a half-center oscillator composed of two mutually inhibitory sets of interneurons, each controlling one of the two dominant phases of locomotion: flexion and extension. To investigate symmetry between the two components of this oscillator, we analyzed the statistics of natural variation in timing during fictive locomotion induced by stimulation of the midbrain locomotor region in the cat. As a complement to previously published analysis of these data focused on burst and cycle durations, we present a new analysis examining the strength of phase locking at the transitions between flexion and extension. Across our sample of nerve pairs, phase locking at the transition from extension to flexion (E to F) is stronger than at the transition from flexion to extension (F to E). This pattern did not reverse when considering bouts of fictive locomotion that were flexor vs. extensor dominated, demonstrating that asymmetric locking at the transitions between phases is dissociable from which phase dominates cycle duration. We also find that the strength of phase locking is correlated with the mean latency between burst offset and burst onset. These results are interpreted in the context of a hypothesis where network inhibition and intrinsic oscillatory mechanisms make distinct contributions to flexor-extensor alternation in half-center networks.

## Introduction

A central debate regarding spinal cord function centers on the question of whether the observed differences between different phases of locomotion result from asymmetric sensory input or from functional asymmetries embedded within the spinal central pattern generator (CPG; [1], [2]). During normal walking, variation in the cycle period is dominated by the ‘stance’ phase, the period of time when the limb contacts the ground. The duration of the ‘swing’ phase, when the limb is in the air, stays relatively invariant. Grillner and Zangger [3], [4] showed that the spinal CPG was capable of producing a rhythm that was qualitatively similar to normal walking even when the sensory inputs and muscle activity were eliminated. Like normal locomotion, the flexor and extensor phases during this ‘fictive locomotion’ were asymmetric, with the flexor phase being relatively invariant in duration, while the extensor phase varied with the cycle period.

More recent investigations into the question of symmetry in the output of spinal CPG, demonstrated that variations in cycle period during fictive locomotion in the cat could be dominated by either flexion or extension [5]. Juvin et al. [6] also showed symmetrical patterns of variation in flexor and extensor burst durations during drug-induced fictive locomotion in the rat. On the other hand, Frigon and Gossard [7] have shown that in the vast majority of spontaneously occurring bouts of cat fictive locomotion are extensor dominated, and have argued that this indicates a strong asymmetry in the structure of the underlying spinal CPG. Recent intracellular recordings from spinal motor neurons in neonatal mice have also shown significant asymmetries between flexor and extensor networks [8].

The starting point for nearly all attempts to understand the functional architecture of the spinal CPG is the half-center oscillator, in which activity alternates between mutually inhibitory subpopulations that control the bursting in flexor and extensor motor neurons. However, rhythmic activity in isolated spinal cords can persist even with inhibition blocked [9], [10], raising the possibility that mutual inhibition may not be the sole mechanism coordinating the transitions between flexion and extension. To examine this coordination, we developed a new statistical measure to quantify the reliability of phase locking at these transitions, and applied this measure to previously published bursting data from fictive locomotion in the cat.

We find that for most nerve pairs phase locking is stronger at extensor to flexor (E to F) versus the flexor to extensor transitions (F to E). These patterns of asymmetry do not reverse for flexor-dominated versus extensor-dominated bouts of locomotion, Furthermore, across nerve pairs the strength of phase locking is systematically related to the mean latency between burst onset and offset. Our approach is complementary to traditional analyses based on burst durations, and demonstrates that phase locking is dissociable from the mechanisms governing the variation in burst durations during MLR-induced fictive locomotion in the cat.

## Materials and Methods

### Ethics statement

This paper presents a new statistical analysis of data from fictive locomotion induced by stimulating the midbrain locomotor region (MLR) of cats. The data were kindly provided to the authors by Dr. David McCrea, were collected as part of previously published experiments independent of this study. Surgical and experimental protocols were in compliance with the guidelines set out by the Canadian Council of Animal Care and the University of Manitoba. Cats were anesthetized using halothane delivered continuously in a mixture of 30% oxygen and 70% nitrous oxide. A precollicular postmammillary decerebration was then performed making the animals insentient (legally dead).

### Experimental procedures

The data analyzed here are from the same set of experiments described and analyszed in Yakevenko et al. [5]. After decerebration, anesthesia was discontinued and the limbs were then paralyzed using gallamine triethiodide (Flaxidel, 2–3 mg per kg-h). Both limbs were extensively (though likely not completely) denervated by bilateral sectioning of the sciatic, femoral, and obturator nerves, and tendons surrounding the hips were cut. Stimulation of the midbrain locomotor region (30–200 mA, 0.5- to 1-ms pulses, 7–30 Hz) elecited activity recorded by placing hindlimb nerves on hook electrodes. ENGs were recorded from up to 12 nerves simultaneously. ENGs received by the current authors had been previously linearly rectified, filtered between 30 hz and 3 khz, low pass filtered at 100 hz and then digitized at 500 hz. Further details about the experimental procedures can be found in Yakevenko et al. [5].

### Data selection and burst detection

Our analysis relies on measuring the statistics of ‘natural variation’ in the timing of burst onsets or offsets relative to the timing of the locomotor cycle. MLR-induced fictive locomotion often contains cycles having deletions, variable burst durations, and inconsistencies in relative timing. To maximize consistency in making these statistical measurements, we only analyzed ‘clean’ stretches of ENG recordings that displayed consistent and regular bursting (defined below). In order to eliminate observer bias we determined burst onset and offset times independently of and prior to the performance of the statistical analysis reported here.

From 21 fictive locomotor recordings obtained, we found 10 bouts of continuous bursting activity that were sufficiently clean, assessed according to the following criteria: 1, Each bout contains a sufficient number of robust continuous cycles (minimum = 17); 2, Bouts show a clear alternation between flexors and extensors and contain no deletions (c.f. [11]; for a discussion on deletions); 3, Bouts have bursts with high slope onsets and offsets with peak activity well above background noise, making them easily detectable using a threshold. Some nerves that were present in the 10 selected bouts were not analyzed for three non-exclusive reasons: there were not enough cycles with consistent bursting in that nerve; the nerve activity had inconsistent phase, i.e. they sometimes were active during flexion, sometimes during extension; or the slopes of burst onset and/or burst offset were too shallow.

Burst onset and offset times were determined by the crossing of a manually determined threshold, set as close to baseline as possible while avoiding baseline noise. In all cases, bursts reported had a consistent shape across bouts. For some nerves like extensor digitorum longus (EDL) whose bursts exhibit a shallow onset and increasing activity over time, noise caused short jumps below and above threshold. To eliminate these, super-threshold crossings less than .05 sec and sub-threshold crossing less than .1 sec apart were discarded. For one nerve in one bout, activity occasionally dipped to near baseline in the midst of a burst. We found that eliminating sub-threshold crossings of up to .4 sec ensured robust segmentation in this case. All analyses were performed using Spikes Show, a custom burst detection program written by Dr. Tim Kiemel, and MATLAB (Mathworks, Natick MA). We have applied similar methods previously within the context of lamprey ENGs [12].

### Data analysis

For consistency with previous studies, we first examined the statistics of burst and cycle durations. The variability in duration was measured using the coefficient of variation, equal to the standard deviation divided by the mean. Correlations between variables was measured using Pearson's correlation coefficient r. To eliminate the contributions from slow drift in variables over the course of a bout, all durations were first detrended by subtracting from each duration *x_i_* the average of all values within a 13 data point window centered on x_i_.

As a complement to traditional analyses focused on burst durations, we examined the phase locking between burst offset and onsets between flexor-extensor nerve pairs at the transitions between phases. For each pairing of a flexor and extensor nerve, we determined the latency from burst offset in the flexor and burst onset in the extensor at the F to E transition and the latency from extensor offset to flexor onset at the E to F transition. Positive latencies correspond to a gap between periods of activation, whereas negative latencies correspond to burst overlaps. We then detrended the vector of latencies using a 13 point moving average as above, and divided by the mean cycle duration for that bout to obtain a list of detrended phase differences θ_i_.

A common measure of the strength of phase locking is the vector strength, defined by averaging the unit vectors (cos(2πθ_i_),sin(2πθ_i_)) and computing the length of the resultant vector [13]. The vector strength (VS) takes on a value between 0 and 1, with 1 corresponding to perfect locking (identical latencies for each cycle) and 0 to random relation between events. However, VS is subject to compressive ceiling effects for nearly synchronous events [14], scaling like the square of standard deviation of phase differences. To obtain a measure that scales more linearly, we paralleled the relationship between VS and angular variance (typically defined as 1-VS), defining the strength of phase locking to be 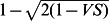, where 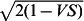 is the angular deviation, one of several measures proposed as the circular analogue of standard deviation [15], [16]. This measure of phase locking is equal to 1 for perfectly locked events and scales linearly away from 1 as the distribution of phases widens.

Unless otherwise specified, values are reported as mean values +/− the standard error of the mean. In a few cases we report mean +/− the standard deviation, which we mark using SD. In many cases we also report the range. Determination of statistical significance is based on two-tailed t-tests for group comparisons. We used the ‘corr’ function in Matlab (Mathworks, Natick MA) to determine the Pearson correlation between latency and phase locking and its significance.

## Results

**Figure 1:**
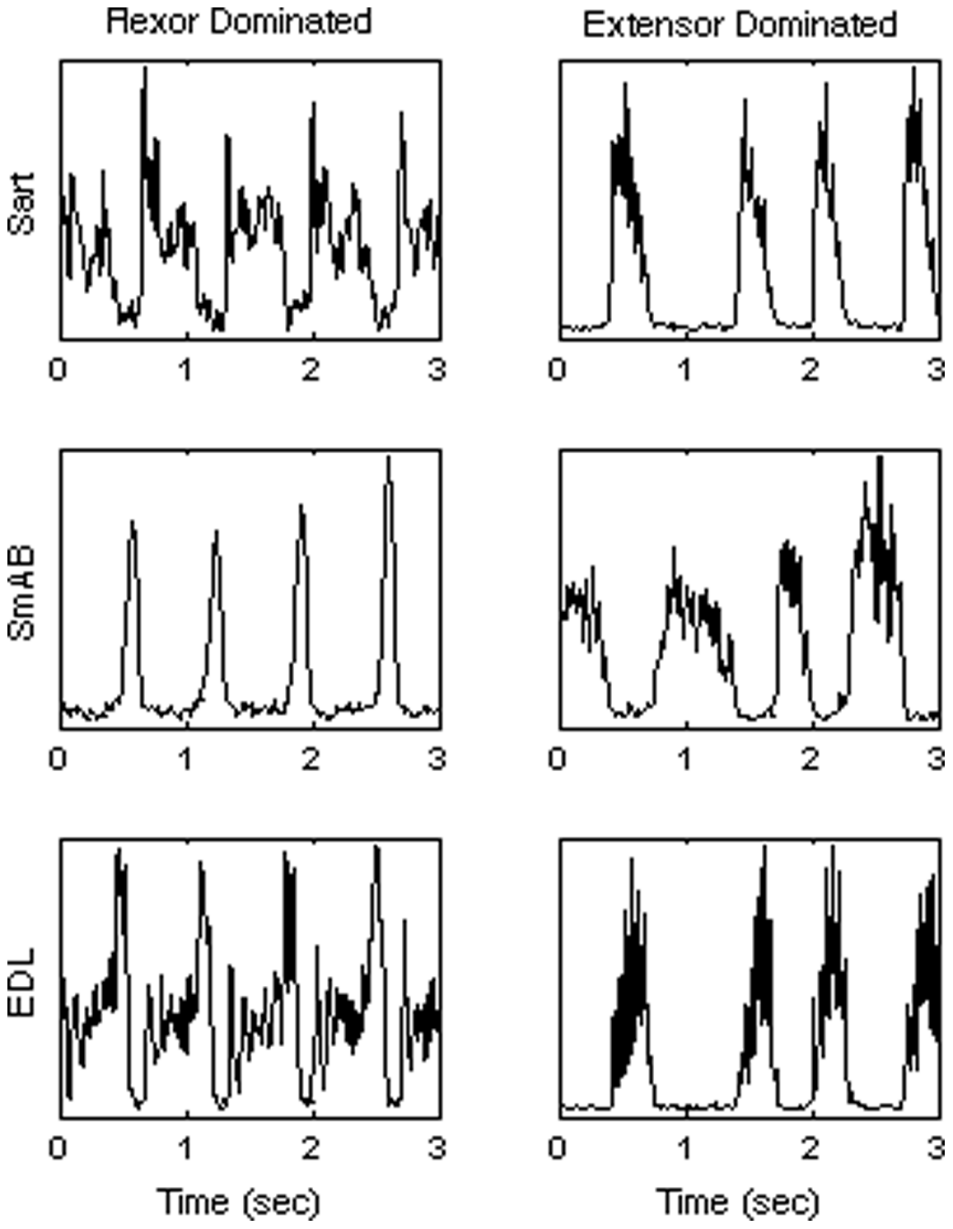
Example traces. Data from three nerves during typical stretches of flexor dominated (left) and extensor dominated (right) fictive locomotion. Shown are Sartorious (Sart), semimembranosus anterior biceps (SMAB), and extensor digitorum longus (EDL).

The output of fictive locomotion can be placed into two broad categories: flexor or extensor dominated. This is defined either by which phase takes up the greater proportion of the cycle [5] or by which phase has a greater correlation with cycle period [3]. Consistent with the results of Yakavenko et al. [5], both definitions yield identical classifications in the data analyzed here. We selected continuous bouts of activity with stable bursting patterns, and analyzed 474 cycles of flexor dominated locomotion from 7 bouts and 157 cycles of extensor dominated locomotion from 3 bouts. Each bout came from a different experimental animal. Example data is shown in figure 1.

Our data set included recordings from the following nerves: the knee flexor sartorious (the lateral head, Sart), the ankle flexors tibialis anterior (TA) and peroneous longus (PerL), the hip extensor semimembranosus anterior biceps (SMAB), the ankle extensors lateral gastrocnemius soleus (LGS) and medial gastrocnemius (MG), and the toe extensor and ankle flexor extensor digitorum longus (EDL), Different sets of nerves were recorded across bouts (Table 1), but all bouts include the flexor Sart and the extensor SMAB.

**Table 1:** Data Summary.

Flexors	Mean Cycle Period	Extensors	Mean Cycle Period
Sart	All bouts	SMAB	All bouts
TA	.639, .814, .907, .854*, .992*, 1.05	LGS	.554, .685, .814, .992*, 1.05
PerL	.521, .814, .854*, .992*	MG	.521, .554, .685
EDL	.521, .639, .736*, .854*, .907, .992*		

List of nerves analyzed sorted by mean cycle period (10 bouts total). All electroneurograms exhibited activity in the flexor Sartorious (Sart), and the extensor semimembranosus anterior biceps (SMAB).

* indicates bout was extensor dominated.

In our data set, EDL was generally co-active with the other flexor nerves, consistent with its role as an ankle flexor. However, statistical analysis of the data revealed that EDL had properties that were unique within this data set. This may relate to EDL's role as a toe extensor as well as an ankle flexor, and is consistent with previous studies showing distinct properties for this nerve [17]–[19]. For simplicity, generic use of the term flexor will refer to the set of flexors in our data excluding EDL, i.e. Sart, PerL, and TA. All analysis that includes the flexor EDL will explicitly refer to EDL by name.

### Statistics of burst durations and cycle periods

Previous statistical surveys of fictive locomotion have focused on the relationship between burst durations and cycle periods [3], [5], [6]. For comparison, we include a short summary of such statistics for our data set. The cycle was defined using burst onset in the flexor Sart, which is included in all bouts in this data set. Variation of cycle period in the 3 extensor dominated bouts (mean detrended *CV* = 24.47±.056%, range 19.47 to 30.61%) is greater than in the 7 flexor dominated bouts (mean 5.58±2.56%, range 2.81 to 9.44%; p = 6.21×10^−5^). Across bout types, extensor bursts are proportionally more variable on average than the flexor bursts (mean detrended *CV* of flexor/extensor bursts = 7.94±0.73/9.28±0.67% respectively during flexor dominated bouts, and 17.79±6.53/36.64±8.02% during extensor dominated bouts).

In 9 of 10 bouts, flexor burst durations were positively correlated with other flexor bursts, and extensor burst durations were positively correlated with other extensor bursts. Flexor pairs (N = 12) had a mean correlation coefficient of .794±.067 (range .369 to .998), and extensor pairs (N = 9) had a mean correlation coefficient of .806±.062 (range .475 to .999). In contrast, correlations between flexor and extensor burst durations (N = 30) are more variable and generally weaker. The mean correlation of a flexor burst with the following extensor burst is −.119±.042 (range −.507 to .511); the mean correlation of an extensor burst with the following flexor burst is −.070±.044 (range −.608 to .309).

In the remaining bout, the correlations between burst lengths in the same phase of the cycle were less consistent. Bursts in two extensor nerves, SMAB and LGS, were negatively correlated (r = −.079) and the correlation between Sart and EDL (r = .338) was the weakest among all flexor pairs examined. Furthermore, this particular bout is unique in that it was the only bout that contained bursting within the posterior biceps-semitendinosis (PBSt) nerve during both the flexion and extension phases of the cycle. Despite these differences, we include this bout in all analyses.

### Phase-locking at transitions between flexion and extension

For the remainder, we adopt a complementary perspective that places a primary focus on the transitions between flexion and extension. For any flexor-extensor nerve pair, we have defined a new measure that quantifies the strength of phase locking between the offset of the previous burst and the onset of the subsequent burst at transitions between phases (see Methods). The difference in timing between these two events is termed the ‘latency’ between burst offset and subsequent onset [4]. Our phase locking measure is based on the distribution of latencies across cycles, and is similar to the commonly used vector strength measure [13] for determining synchrony. However, our measure avoids the compressive nonlinearity of the vector strength measure when phase locking approaches the maximum value of one.

Across all nerve pairs, mean latencies were 21.84±28.84SD msec (range = −40.82 to 80.77 msec). These values are roughly five times larger than the longest expected propagation time from spinal motor neurons to distal ENG recording sites. Spikes propagating at the relatively slow rate of 70 m/s [20], [21] will arrive 0.3 m distant from the spinal cord with a latency of 4.3 msec. Variations in this timing will likely be less than 20% of the mean propagation time or less than one millisecond. In contrast, the distribution of standard deviations of individual ENG latencies was 12.62±7.10SD msec (range = 4.52 to 34.72 msec). This suggests that the variations in ENG burst latencies are dominated by variations in the timing of activity within motor neurons driven by the spinal CPG.

**Figure 2:**
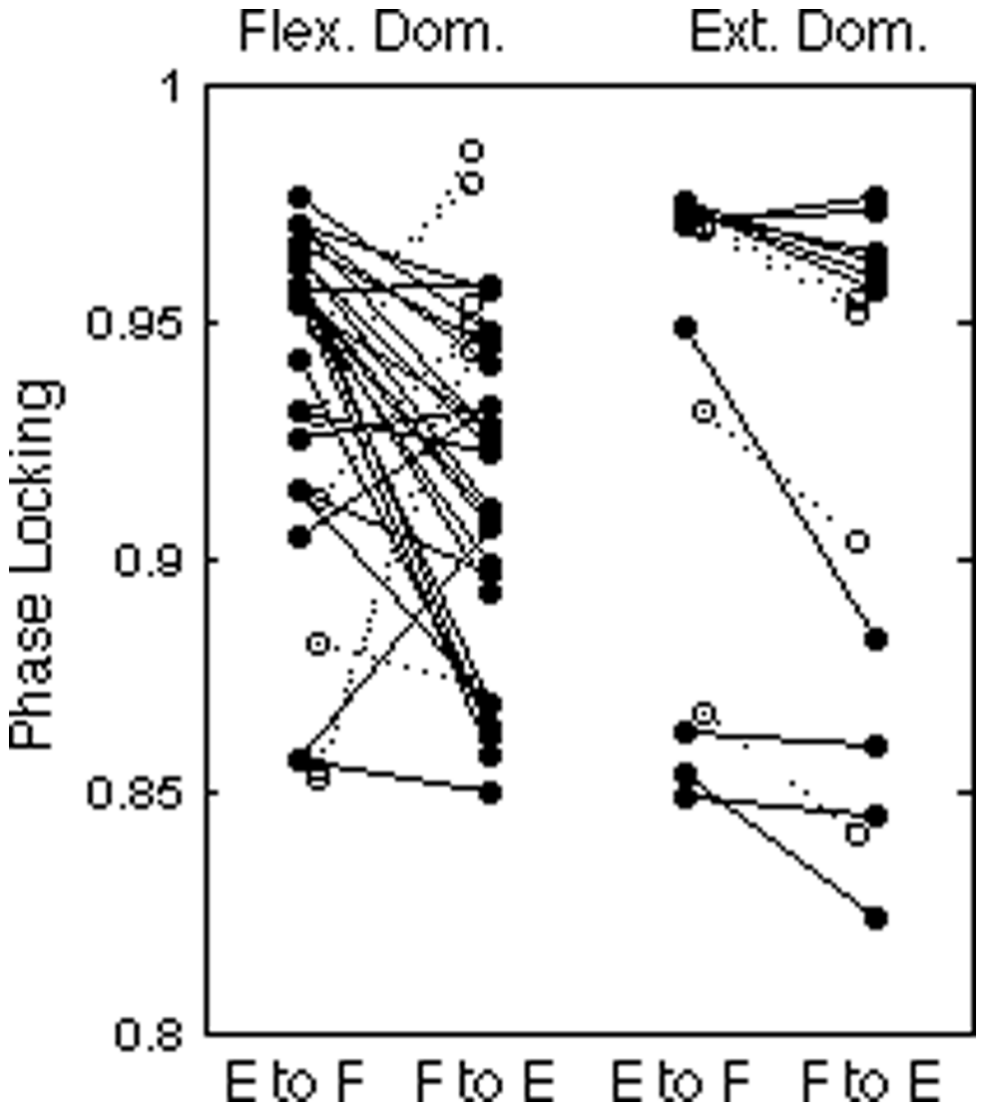
Asymmetric phase locking at burst transitions. Each nerve pair is depicted as a line connecting the strength of phase locking at the E to F transition (left) and at the F to E transition (right). Flexor dominated bouts are shown at left; extensor dominated bouts at right. Solid lines show that for most nerves, phase locking is weaker at the F to E transition. Phase locking between EDL and the extensors SMAB, LGS, and MG (open circles, dashed lines) shows a similar trend for extensor dominated bouts, but the opposite trend for flexor dominated bouts.

Applying our phase locking measure to the data, we find that the strength of phase locking between flexors and extensors are strongly asymmetric, with the transition from extension to flexion (E to F) more tightly phase locked than the transition from flexion to extension (F to E; figure 2). Stronger locking at the E to F transition is found for 28 out of 34 nerve pairings, with E to F minus F to E strength averaging ..0288±.0058 (p = 2.15×10^−5^). This asymmetry is highly significant for the 24 pairings in flexor dominated bouts considered alone (average difference .0346±.0075; 20/24>0; p = 6.68×10^−6^). For the 10 pairings in extensor-dominated bouts the asymmetry was in the same direction, with the difference approaching the edge of statistical significance (average difference .0149±.0066; 8/10>0; range *p* = .0505).

The flexor EDL appears to be different from the other flexors (figure 2, open circles). Although the numbers are small, 5/6 pairings including EDL in flexor dominated bouts showed stronger phase locking at the F to E transition (mean difference −.0504±.0163; p = .0271) whereas locking with EDL in 4/4 pairings within extensor dominated bouts showed tighter phase locking at the F to E transition (mean difference .0219±.0029; p = 4.82×10^−3^).

This pattern of phase locking asymmetry was consistent across our data set, with stronger phase locking as the E to F transition versus the F to E transition in at least 70% of the cases for each nerve pair considered separately (number a bouts ranging from 2–10 across pairs; figure S1). The difference in EDL phase locking for flexor dominated versus extensor dominated bouts was also seen in both pairings of EDL with an extensor where we had data from both flexor dominated versus extensor dominated bouts (figure S1).

### Latencies and phase locking at transitions

The time of subsequent burst onset minus the time of previous burst offset at the given transition is termed the ‘latency’ between burst offset and subsequent onset [4]. In examining the data, we noticed that there was often a short period of overlap (negative latency) between bursts at the E to F transition, whereas latencies were generally positive at the F to E transition. The mean latency at E to F was 6.66±26.25SD msec (range −40.82 to 70.20 msec); mean latency at F to E was 37.03±22.91SD msec (range = .27 to 80.77 msec). As expected from the asymmetry in the phase locking measure, the variability in latency was also asymmetric; the standard deviation of latency at E to F was 10.26±6.95SD msec (range 4.52 to 30.05 msec), and was 14.97±6.54SD msec (range = 5.38 to 34.72 msec) at F to E.

When grouping data across bouts, we express latency as a fraction of mean cycle duration, Using this normalized measure, the mean latency at E to F was 1.03±0.57SD % (range −5.80 to 6.68%); mean latency at F to E was 4.88±0.58 SD % (range = 0.05 to 12.88%). The E to F minus F to E latency is significantly negative across nerve pairs (mean = −3.85±0.70%; range −10.95 to 5.52%; *p* = 5.04×10^−6^), and holds separately for flexor dominated bouts (mean difference = −3.89±0.98%; range −10.95 to 5.52%; *p* = 6.26×10^−5^) as well as for extensor dominated bouts (mean difference = −3.75±0.56%; range −8.12 to −2.05%; p = 8.57×10^−5^).

**Figure 3:**
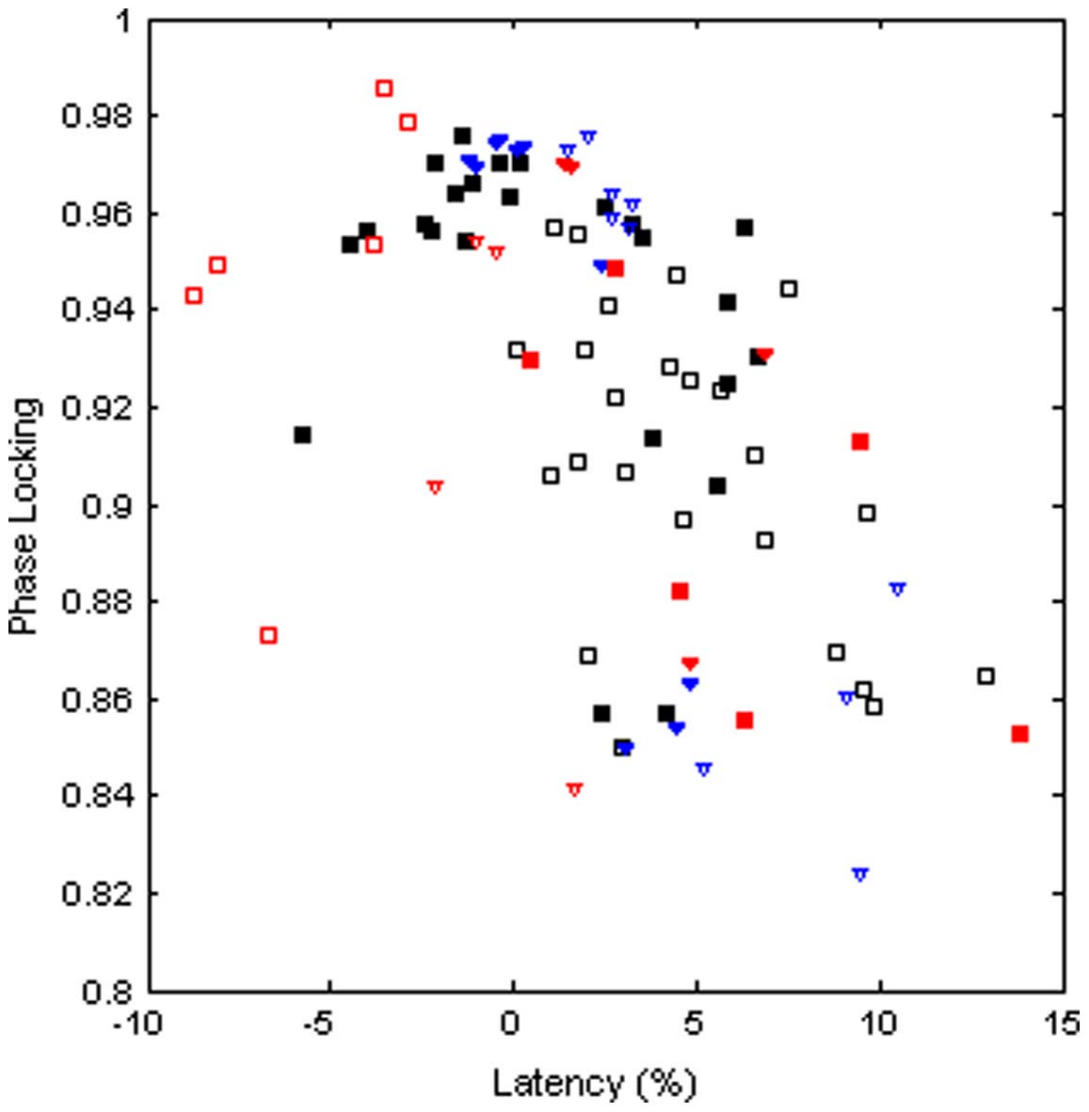
Phase-locking and burst latency. Increasing latency between onset and offset of flexor and extensor bursts is correlated with decreased strength of phase locking. The correlation is significant for extensor dominated bouts (blue triangles) as well as flexor dominated bouts (black squares). For most nerve pairs, mean latencies tend to be positive and large at the F to E transition (open symbols), and small or negative at E to F (solid symbols). The negative correlation between latency and phase locking also holds for the flexor EDL (red; triangles/squares show extensor/flexor domination; open/solid symbols show F to E/F to E transitions).

The fact that latencies are shorter and the phase locking is tighter at the E to F vs. the F to E transition naturally leads to a negative correlation between latency and phase locking (fig. 3; linear regression, *r* = −.620, *p* = 1.76×10^−8^). Less obviously, the negative relationship between latency and phase locking persists when each transition is considered separately (F to E transitions, *r* = −.629, *p* = 6.73×10^−5^; E to F transitions, *r* = −.474, *p* = 4.62×10^−3^). Therefore, the relationship between latency and the strength of phase locking is not a simple consequence of the E to F vs. F to E asymmetry. Note also that the strength of phase locking cannot be simply explained by the absolute distance between events, since phase locking does not become consistently weaker with increasingly negative latencies.

As has been shown previously [17]–[19], the latency of EDL is distinct from the other flexors during both intact and fictive locomotion. The offsets of EDL bursts at the F to E transition tend to occur after the onset of extensor bursts (mean latency = −3.58±1.07%, range = −8.06% to 1.65%). Conversely, EDL onset tends to occur later than extensor offset at the E to F transition (mean latency = 5.20±1.31%, range = .44 to 13.88%). These differences between EDL and other flexors are significant (*p* = 2.07×10^−3^ for E to F latencies; *p* = 1.57×10^−8^ for F to E).

Although EDL differs from other flexors both in the strength of phase locking and its latency with extensor bursts, EDL phase locking and latency are negatively correlated, consistent with the relationship observed across all bouts and nerve pairs (*r* = −.468, *p* = .0373; figure 3, red symbols). This consistency argues that the factors determining the mean latency are closely related to those that determine phase locking at transitions.

As with phase locking asymmetry, the general relationship between phase locking and mean latency held for nerve pairs when considered separately, including pairing with EDL (figure S2).

## Discussion

Early on, it was hypothesized that the spinal cord contains an ‘intrinsic factor’ or central pattern generator (CPG) composed of two mutually inhibitory sets of interneurons, each associated with either flexion or extension [1], [22]. This circuit is known as a half-center oscillator. Initially it was thought that these two sets of neurons were functionally identical, with the observed differences in flexor and extensor activity being caused by differences in sensory inputs. Several studies have examined this issue by quantifying patterns of variation in burst durations during fictive locomotion, with some authors favoring the hypothesis that there exist structural asymmetries within the spinal CPG [3], [4], [7], [23], [24] and others maintaining that the differences between extensor and flexor burst durations arise from asymmetric control signals impinging upon an essentially symmetric CPG [5], [6].

As a complement to measuring burst durations, we have analyzed the strength of phase locking between onsets and offsets of ENG bursts at the transitions between the two basic phases of locomotion. We find that during MLR-induced fictive locomotion in the cat, the phase locking is consistently stronger at the transitions from extension to flexion (E to F) than at the transition from flexion to extension (F to E). This difference is found regardless of whether flexor or extensor phase of the cycle was dominant, suggesting that the mechanisms determining the dominance of burst durations and strength of phase locking are dissociable.

Across transitions, we find that the phase locking is positively correlated with the latency between burst offset and subsequent onset, with burst overlap correlated with tight phase locking and large latencies corresponding to loose locking. The robustness of the relationship between mean latency and phase locking is illustrated by the exceptional characteristics of the flexor EDL. Bursting in EDL is delayed relative to the other flexors examined here (Sart, TA, and PerL), leading to longer latencies at the E to F transition and shorter or more negative latencies at the transition from F to E. EDL also shows differences in phase locking, with opposite patterns for extensor and flexor dominated fictive locomotion (fig. 2). These results corroborate previous suggestions, based on differential response to stimulation of the periphery [25], [26], that EDL is controlled by distinct circuitry. Despite these differences, the strength of phase locking between EDL and other extensors follows the overall negative relationship with mean burst latency found for other nerves.

### Phase locking in half-center oscillators

Although most models rely on reciprocal inhibition to drive the alternation between the two basic phases of the step cycle, it has been shown that rhythmic activity persists within spinal cords even when inhibition has been blocked [9], [10]. Under the assumption that each of the two subpopulations in the half-center circuit is able to oscillate when isolated from the network, there are then two mechanisms that can contribute to the timing of burst onsets and offsets: the oscillatory mechanisms intrinsic to the component oscillators and the network inhibition between the opposing oscillators for flexion and extension.

These mechanisms are expected to lead to different patterns of phase locking at the transitions between flexor and extensor phases. As an illustration, consider the case where the component oscillators of the half-center are active for less than 50% of their cycle period when decoupled from any network, i.e. their ‘duty cycle’ is less than 50%. With mutual inhibition, we expect relatively minor changes in their behavior, since as long as their active periods do not overlap, transitions between the active and inactive states occur when the other oscillator is inactive. Therefore, the exact timing of transitions would be largely determined by dynamics internal to each oscillator, leading to weak phase locking.

Now suppose that inputs to the system would cause an isolated oscillator to have a duty cycle greater than 50%. Then the burst of one oscillator would be too long to ‘fit’ into the off period of the other oscillator. In the coupled network, the onset of bursting would lead to active inhibition of the other oscillator. In particular, network inhibition would link the timing of burst onset in one oscillator with burst offset in the other oscillator, causing the two to become phase locked.

We suggest that the differences in phase locking reported here may reflect some functional asymmetry in the underlying CPG that lead to latencies that are shorter at the E to F than at F to E transition. This causes the E to F transition to be more strongly dominated by mutual inhibition, resulting in tighter phase locking.

### Factors affecting transitions in oscillator models

Previous research has shown that half-center oscillators operating with strong network inhibition can further be subdivided according to whether the transitions are initiated by an ‘escape’ of the inactive neuron from suppression, or burst cessation in the active neuron that then ‘releases’ the inactive neuron from inhibition [27]–[30]. The notions of escape and release are most clear in the case of strong inhibition, and so we would expect models relying on either mechanism to display tight phase locking at burst transitions.

Rybak et. al. [31] have extended the basic half-center model, proposing that the spinal locomotor network is composed of two functionally distinct sets of interneurons: a set of high level ‘rhythm generator’ neurons sets the overall speed of locomotion and the gross duration of flexion and extension, while a second layer of premotor ‘pattern generator’ interneurons shapes the activity in individual muscles or muscle groups [11], [25], [32]. The timing of burst onset or offset in any given nerve could be due to inhibition arising from either or both levels of this hierarchical network, as well as from oscillatory mechanisms intrinsic to the neurons driving activity in that nerve. While the data set we have analyzed here is insufficient to place strong constraints on models of this complexity, phase locking at transitions between flexion and extension may be a useful indicator of how closely linked a given set of motor neurons is to the output at different levels of the CPG hierarchy.

## Conclusions

We have analyzed the strength of phase locking between burst timing at the transitions between extension and flexion during MLR-induced fictive locomotion in the cat. These results are complementary to a previous analysis of the same data set based on burst durations, and suggest that asymmetries in the strength of phase locking are determined by circuit mechanisms and/or experimental parameters distinct from those governing asymmetries in the duration of flexion and extension. This paper analyzes data from a single set of experiments in which the inputs to the system were not systematically varied. By measuring changes in phase locking across a variety of tasks and experimental conditions it may be possible to uncover important state-dependent differences in network function that are induced by experiment, injury or disease.

## Supporting Information

Figure S1 **Phase locking asymmetry by nerve-pair.** Each line connects the strength of phase locking at the E to F transition (left) and at the F to E transition (right) for a single nerve pair in a single bout. Flexor dominated bouts are shown with circles; extensor dominated bouts with triangles. Stronger phase locking at the E to F transition is found for each flexor-extensor nerve pairing considered separately. For both SMAB, and LGS, phase locking with EDL is stronger phase locking at E to F for flexor dominated bouts and weaker locking at E to F for extensor dominated bouts.
(TIFF)

Figure S2 **Mean vs. standard deviation of raw latencies by nerve-pair.** Each line connects the mean latency and standard deviation of the latency for a single nerve pair in a single bout. E to F transitions are shown with closed symbols and F to E transitions with open symbols. Flexor dominated bouts are shown with circles; extensor dominated bouts with triangles. Since smaller standard deviations correspond to stronger phase locking, the overall trend of having stronger locking with lower mean latencies (lines with positive slope) is followed for most nerve pairings. The only consistent exception is for pairings with EDL in extensor dominated bouts, which shows the opposite trend.
(TIFF)
